# The Holocaust and Disciplinary Myopia in Criminology and Sociology: Social injury as a response to the challenges of legal formalism

**DOI:** 10.1007/s10611-022-10031-4

**Published:** 2022-05-27

**Authors:** Douglas J. Dallier

**Affiliations:** Department of Sociology, Criminal Justice, and Geography, Winona State University, Winona, MN 55987 USA

## Abstract

Criminological and sociological discourse recognizes the impact of structure on crime, but generally eschews the consideration of structural damage and human suffering emanating from malevolent social movements (e.g., the Holocaust). Legal formalism presents conceptual challenges that has hindered analysis of harmful macroscopic phenomena, as it created jurisprudential impediments to be surmounted by the architects of the Nuremberg Tribunals. In considering these issues, a new ‘dark figure’ is identified that is compatible with phenomena examined from the social harm perspective, and to remediate disciplinary myopia, a specification of Edwin Sutherland’s (1945) concept of social injury is suggested and contrasted with Galtung’s (1969) construct of structural violence. Social injury refers to the recursive damage to social structure and human potential through the functional impairment of social institutions.

In his examination of the systematic persecution of indigenous peoples, Chris Cunneen questioned the suitability of criminological discourse to contribute meaningfully to an examination of events where responsibility is not solely attributable to individuals (2016). To Cunneen, a focus on subjects like state crime can challenge the limits of existing criminal codes that are best suited to determinations of individual responsibility. This conundrum was faced by those entrusted with developing the London Charter, which served as a legal framework for the prosecution of atrocities committed in the Holocaust. Such organizational behavior additionally challenges the sociological concepts of ‘normal’ and ‘deviant’, eliciting inquiry about the contexts under which those labels can and have been applied and disclosing the (often indirectly) politicized nature of sociological and criminological discourse on crime. Cunneen questioned “to what extent was criminology actually complicit in providing a scientific foundation for taking Indigenous children from their families and institutionalizing them, and to what extent does it continue to do so today? In other words, we need to consider whether criminology had and has a stake in genocide” (2016, p. 137). This article does not consider that important subject- the role that sociological and criminological discourse played in the perpetuation of genocide; rather, it addresses the spirit of Cunneen’s lament by examining discursive gaps in sociology and criminology, to understand the factors that inhibit systematic analysis of phenomena not easily comprehensible within the context of codified law (i.e., genocide). This article circumscribes an expanded ‘dark figure’ and suggests an epistemic solution to disciplinary myopia by respecifying Edwin Sutherland’s (1945) construct of social injury.

## The case for the Holocaust and taciturn sociology

Sociology and its subdiscipline, criminology, have examined the impact of power differentials on many aspects of social life. Within those disciplines exists a more discrete discourse focusing on the contributors to harms as disparate as: the human rights abuses committed by the resource extraction industry in Papua New Guinea (Lasslett, 2014); the profiteering and regulatory incompetence contributing to the Challenger space shuttle explosion (Kramer, 2006; Vaughan, 1996); politically and economically rationalized environmental destruction (Kramer, 2020; Kauzlarich & Kramer, 2006); longstanding dislocations and unrest in the Middle East stemming from the Balfour Declaration of 1917 (Falk, 2019); and the inequalities perpetuated and magnified by the criminal justice system itself (Reiman & Leighton, 2017). Despite the consideration of disparate types of injustice, entire categories of phenomena challenge the applicability of existing sociological and criminological constructs. The Holocaust, genocide, and harms perpetuated by the state have remained largely distal from the corpus of research in these disciplines that one might presume would provide insightful critical analysis.

Even though the term ‘genocide’—the systematic attempt to destroy a people or their culture—first appeared in print more than 70 years ago (Lemkin, 1944), it is striking (and somewhat notorious) that these events have been largely overlooked by sociologists and criminologists (Bauman, 1988, 2000; Day & Vandiver, 2000; Friedrichs, 2000; Hagan & Rymond-Richmond, 2009; Maier-Katkin et al., 2009; Rafter, 2016; Yacoubian, 2000). Despite a few articles scattered throughout the intervening years, this discursive gap is paradoxical given genocide’s enormous impact on society, made obvious in the scholarship on the *Final Solution to the Jewish Question* (Moore & Muller, 2002). Zygmunt Bauman concluded that sociology’s general myopia with regard to understanding the Holocaust was established “beyond reasonable doubt”, famously lamenting that “*the Holocaust has more to say about the state of sociology than sociology in its present shape is able to add to our knowledge of the Holocaust*” (1988, p. 471; 2000; emphasis in original). The relevance of the Holocaust to its subdiscipline, criminology, was particularly acute to David O. Friedriche:

“The combination of the events of the Holocaust, the response to them, the massive literature and analysis, the Holocaust's role as metaphor, the impact and influence of the event - that is, the totality of the event itself and its aftermath - renders the Holocaust a *criminal event* apart from all others” (2000, p. 22-23; emphasis added).

Friedriche’s reference to the Holocaust as a “criminal event” is, however, not without its conceptual challenges, some of which inhibit criminological analysis. Despite Rafter’s (2016) behavioral analysis of eight genocidal events, criminologists have yet to contend with many of the legal-philosophical dimensions of genocide. Additionally, the nature of these events (and those who perpetrated them) challenge sociological concepts like ‘deviant’ and tests the moral boundaries of structural functionalism, since many contributors have been disconcertingly characterized by their conformity in addition to operating in accordance with the law (at that time). The scholarship on normalcy is expansive, from Raul Hilberg’s *Destruction of the European Jews* (1985) to Hannah Arendt’s *Eichmann in Jerusalem* (1963) to Haim Gouri’s *Death Dealer* (2004) to Christopher Browning’s *Ordinary Men* (1998), all illuminate the banal bureaucratic machinery that facilitated Fordist killing. The extent of harm emanating from the Holocaust (one of the most malevolent orchestrated undertakings in modern human history) makes the paucity of scholarship in sociology and criminology profoundly paradoxical (Afflitto, 2000; Bauman, 1988, 2000; Chambliss, 1995; Day & Vandiver, 2000; Eliot, 1972; Friedrichs, 2000; Hagan et al., 2005; Hagan & Rymond-Richmond, 2009; Hoffman, 2000; Levene, 2000; Maier-Katkin et al., 2009; Mares, 2009; Presser, 2013; Rafter, 1990, 2016; Steiner, 2000; Welch, 2009; Yacoubian, 2000).

Although frequently referred to as a singular event through the clarity of hindsight (Lemkin, 1944; Rubenstein, 1978), the many piecemeal actions that facilitated the Holocaust reveals social dynamics of paradigmatic significance for sociology and criminology. The criminal trials of several organizations and individuals involved in perpetuating the Holocaust highlights both this broad scope of complicity and the challenges inherent to identifying, codifying, and holding accountable the behavior of those involved (Seibel, 2005). Malevolent social movements like the Holocaust require broad spheres of action and complicity, blurring the lines that circumscribe culpability. Benjamin Ferencz, a prosecutor at the Nuremberg Tribunals, remembered “it being quite obvious to us that the killing of six million people and the commission of all the other atrocities could not be accomplished without a conspiracy implicating the entire German administration and apparatus” (1990, p. 327). Similarly, Browning (1983) noted that “the Nazis’ mass murder of the European Jews was not only the technological achievement of an industrial society, but also the organizational achievement of a bureaucratic society” (p. 149). Scholarship on the Holocaust also suggests that modern organizations and bureaucracies do not necessarily serve as bulwarks against inhumanity, as is sometimes implied when juxtaposed with the ‘primitive’ societies thought to characterize humanity’s more brutal ancestral past. This was implicit in Stanley Milgram’s (perhaps infamous) studies on obedience- that humans are generally capable of impersonally harming others with little provocation despite antecedent structural conditions thought to guard against such behavior (e.g., education; 1974). The existing scholarship suggests that it is perhaps more accurate to say that modern forms of social organization (e.g., bureaucracy) can be, and have been, used in the organization and implementation of a wide variety of human activities.

Aside from challenging ascriptions of culpability, sociological examination of the Holocaust holds the potential of expanding our understanding of the nefarious implications of progress. Consider that, while there have been many pogroms and persecutions throughout history, they are frequently associated with the least educated, least economically and technologically advanced societies; see, for example, Hagan and Rymond-Richmond’s (2009) study on the genocide in Darfur, a region which could be characterized as such. In contrast, analysis of the Holocaust illuminates the dystopian potential of modern, ‘advanced’ society, since Germany was at the apex of the intellectual world at that time (Bauman, 1988); an abundance of intellectual capital provided no bulwark against genocide (quite the opposite, after physicians had perfected the technology to eliminate “useless eaters”; Rubenstein, 1978). Additionally, the Holocaust perhaps represents the furthest ends served by categorizing, dehumanizing and discriminating amongst people (Allport, 1954). In this sense, it serves as a powerful warning of the potential stemming from nefarious political agendas, particularly when buttressed by a discourse aimed at establishing the existence of the ‘criminal mind’ or its juxtaposition with one’s biological constitution.

The Holocaust also problematizes the sociological concept of ‘deviant’ and the extent to which it embodies pathology (Gibbs, 1966). The volume of organizational actors facilitating the Holocaust (Hilberg, 1985) makes employing that term (‘deviant’) as thorny as establishing legal culpability, since going *against* the social forces that facilitated Fordist death would certainly have constituted deviance (despite no historical evidence indicating coercive threats were ever necessary; Browning, 1998). Rather than constituting ‘deviance’, the actions that brought about the Holocaust were *representative* of social order; thus, they did not signify aberration, instead exemplifying German social organization (Aly & Heim, 2002; Hilberg, 1985; Lankford, 2009; Rubenstein & Roth, 1987; Waller, 2007). Vaughan (1996) characterized this general phenomenon as the “normalization of deviance” (p. 409), where harmful or injurious activities begin innocuously then incrementally increase in intensity without arousing the awareness of participants, important in understanding the most banal (yet essential) contributors to the Holocaust. In light of these considerations, it is perhaps unsurprising that the conformity in the Holocaust has yet to be reconciled with many existing sociological and criminological theories concerned with malevolent (sometimes framed as ‘deviant’) behavior. Cunneen viewed this conundrum as signifying the need for a “dramatic rethinking” of the concepts of normality and deviance (2016, p. 125).

## The London Charter

Asserting that the Holocaust was perpetrated largely by those working from within organizations obfuscates the historical fact that many individuals persecuted Jews and others without such assistance, making it even more striking that contemporary criminology and sociology have remained aloof from understanding contributory social dynamics. This aloofness comports with the jurisprudential challenges faced in applying the construct of ‘crime’ to these atrocities, as was the case at the close of World War II, when criminal culpability was only cautiously ascribed to a few (initially only twenty-four individuals and seven organizations) deemed legally blamable for facilitating the *Final Solution*. Agreed to by twenty-three nations on August 8, 1945 (Wechsler, 2013), adjudicatory efforts were targeted at the most powerful and influential members of the German state; in essence, those that would present the least difficulty in establishing a successful case at trial (Mitgang, 1992).

The London Charter served as legal justification for the military tribunals at Nuremberg, setting forth the charges to be levied. As Ferencz recalled, arriving at its rationale was a complicated affair, because “the British, for example, who always were noted for their fair play, were in favor of just taking them out and shooting them” (1990, p. 326). The London Charter marked a significant historical turning point; for the first time, nations could be held legally accountable to other nations for actions carried out within their own jurisdictions (and thus not defined as crime), made possible through the creation of a new crime type: “crimes against humanity” (Ferencz, 1990). Expectedly, challenges to drafting the Charter were formidable, resulting from concerns over the enactment of *ex post facto* law (Wasserstrom, 1971) in addition to the limits of circumscribing culpability- deeply problematic given the breadth of knowledge about, and complicity with, the *National Socialist* agenda (Falk, 2019). Herbert Wechsler (2013) noted the limitations built into this novel legal construct:

“The definition of crimes against humanity supplemented that of crimes of war — but the concept was accorded very little scope because of the requirement that the acts of inhumanity included be committed “in execution of or in connection with” some other crime within the jurisdiction of the Tribunal, that is, a crime against peace or war crime” (p. 140).

In contrast to the paucity of subsequent sociological and criminological analysis, a relationship between genocide and social structure was apparent to the planners of the Nuremberg Tribunals; the sheer scope of participation in the *Final Solution* undermined the practicality of holding vast swaths of the German population accountable, and was even perhaps Quixotic, lest they further destabilize an already tenuous European society (Taylor, 1992). Despite challenges, idealism underscored the importance of a just response, for, according to Ferencz: “if we could establish, as a rule of law, the right of all human beings to live in peace and dignity regardless of their race, regardless of their political opinion or ideology, such a principle would lead to a more humane and peaceful world” (1990, p. 328). Given the jurisprudential impediments, it is unsurprising that formal legal definitions of crime present epistemic impediments to systematic criminological and sociological analysis of phenomena that victimizes on the scale of nation states.

## Legal formalism and taciturn criminology

The widespread participation necessary for facilitating the Holocaust calls into question the general applicability of specific legal constructs relevant to criminological discourse (e.g., *mens rea*, *actus rea*, *mala in se* and ascriptions of criminal culpability). Hannah Arendt (1963) disconcertingly observed that Adolf Eichmann—convicted and executed former Nazi bureaucrat who managed Jewish affairs—represents a “new type of criminal” who commits atrocity under circumstances that make it impossible for them to know (or feel) that they were doing wrong (p. 276). Although a seemingly dubious proposition, Arendt suggested that careerists like Eichmann went about their business without fully contemplating the connection between their individual actions and the moral implications of larger organizational outcomes; her argument becomes much less dubious when considering the piecemeal contributions of lower-level actors. In modern criminal law, preclusion of the formation of *mens rea* (criminal intent) typically entails freedom from legal culpability, a condition that Arendt saw as particularly troublesome, since “the problem with Eichmann” is that he was seemingly so normal, and that there were so many like him (Arendt, 1963, p. 276). If one moves beyond Eichmann and considers the multitudes of otherwise normal individuals who had a piecemeal role in facilitating the *Final Solution*—most of whom were never identified, much less held accountable—the possibility of establishing criminal culpability becomes inconceivable, a dilemma familiar to the draftees of the London Charter (Taylor, 1992).

Even the construct of *actus rea* (criminal action itself, independent of intent) is presented with conceptual challenges. For, while it took merely a single individual to dump a canister of *Zyklon B* into the gas chambers at Auschwitz (murdering multitudes within), it took many thousands of individual and organizational actors to relocate the victims to that specific place at that particular time: engineers, architects, population experts, travel agents, financiers, to name but a few (Aly & Heim, 2002; Hilberg, 1985; Rubenstein & Roth, 1987). Haim Gouri’s (2004) observation that “tens of thousands of murderers of all ranks, uniforms and departments are scattered across Germany, wearing civilian clothes” (p. 154) led him to chillingly conclude that “there were more PhD’s who had a hand in the destruction of the Jews of Europe than there were sergeants in the *einsatzcommando*”, a reference to the motorized units directly involved in murdering Jewish people before physicians and industrial engineers perfected a more impersonal—and industrially efficient—process (p. 161; emphasis in original). The macroscopic scale of malevolent social movements makes circumscribing the boundaries of participation (and hence culpability) difficult. In addition to challenging concepts central to definitions of crime (*mens* and *actus rea*), a further conundrum lie in that the Holocaust was perpetrated by multitudes of normal, educated, conforming people who proved themselves fully capable of contributing to murder on a societal scale, despite the presence of factors relevant to understanding desistance in many contemporary criminological theories (e.g., familial bonds, educational attainment, employment).

These considerations highlight a paradox whereby the social dynamics that facilitated the Holocaust simultaneously represent significant phenomenon meriting analysis yet remain largely unaddressed in the criminological literature. As has been argued, this is in part because many of the behaviors that contributed to the *Final Solution* were conforming in their time, and not in violation of codified law (Aly & Heim, 2002; Maier-Katkin et al., 2009; Rubenstein & Roth, 1987). Robert Agnew (2012) attempted to transcend the limitations of formal definitions of crime (legal formalism) with the concept of “blameworthy harms”, defined as “those for which individuals or groups bear some responsibility, are unjustified, and are inexcusable” (p. 25). Despite an expansive formulation that addresses some limitations, Agnew’s concept is challenged when considering the Holocaust, because hierarchy and a widespread division-of-labor obfuscated the degree of requisite “voluntariness and intentionality” for piecemeal organizational participants (2012, p. 25). Organizational and structural constraints (e.g., hierarchy, capitalism, social norms, etc.) impact the free imposition of the will, calling into question post hoc determinations of responsibility for low-level contributors (Bandura, 2004). Additionally, the larger the organizational undertaking, the more obscured overall objectives may be, particularly for those distal from decision making power; the role of taxpayers in facilitating the *Final Solution* illustrates this. Agnew’s concept of blameworthy harms is further challenged by the requirement they be “unjustified and inexcusable” (p. 25), problematic when the state itself engages in both perpetuating harm and circumscribing permissible justifications; necessity and self-defense are often referenced as part of the misguided logic invoked to justify systematic murder in the Holocaust. Furthermore, Agnew’s formulation of blameworthy harms opens the door for the perpetuation of state harms so long as some justification serve as a pretense, undermining the basic human right to self-preservation (existence) and creating conditions that allow for the reification of Churchill’s caution about victors crafting self-serving historical narratives.

Seemingly banal legal constructs like *mens rea* and *actus rea* impede criminological analysis of the Holocaust, as they challenged framers of the London Charter. Likewise, formulations explicitly created to transcend legal formalism can prove troublesome when considering malevolent social movements. There are many factors that have been implicated in disciplinary myopia- the priorities of state research sponsorship, a culture within criminology oriented towards examination of ‘traditional’ crimes, the neoliberal commodification of education, a tenure and promotion process that rewards efficiency (Tombs & Whyte, 2003). As this paper suggests, myopia is also partly related to the implicitly agnostic stance legal formalism takes regarding the role power differentials play in legislatively differentiating unwarranted from acceptable behavior, of great interest to scholars working from within the social harm perspective.

## Legal formalism and social harm

Scholars in the critical tradition have evaluated the impact of formal legal definitions on discourse ostensibly aimed at examining harmful phenomena (Faust & Carlson, 2011; Hillyard & Tombs, 2007; Lasslett, 2010; Michalowski, 2009; Pemberton, 2007; Quinney, 1970; Schwendinger & Schwendinger, 1970, 1972). Thorsten Sellin cautioned that criminology could not be considered analytic unless it eliminated “its meta-theoretical practice of allowing the power structures that underlie positive law to determine the subject matter of the field” (in: Michalowski, 2009, p. 307; see also: Sellin, 1939). Similarly, Schwendinger & Schwendinger (1970) contended that “all that remains of legalistic definitions is the formal acquiescence, on the part of researchers, to such conceptual categories as robbery, rape or homicide” such that “political power determines the precision of the definitions and the measurement of the phenomena” (p. 132–133). Falk’s (2019) analysis of international law noted this Churchillian characteristic- that equivalent phenomena can be defined as ‘political necessity’ or ‘barbarity’, depending upon whether one is situated on the winning or losing side of conflict. Hillyard and Tombs (2007) stress that the construct of ‘crime’ not only *distracts* from more serious social harms unconsidered by formal definitions, but sometimes *excludes* more serious harms from analysis. Cunneen (2016) similarly noted that legal formalism creates a type of disciplinary hypocrisy, in that “the issue of human rights abuses goes to the heart of many questions which criminology has had so much to say about in the case of individual behaviour, but so little to say about when it comes to large scale organised and legitimised criminal behaviour by state agencies” (p. 137). The epistemic implications of distraction and exclusion are significant, as Michalowski (2009) cautioned: “limiting criminological analyses to only those harmful actions that have been prohibited through the workings of the hegemonic power structures inherent in law formation renders criminological inquiry constitutive of the dominant social order rather than analyses of that order”, and he considered this the “most significant factor placing the power crimes of empire outside the consciousness of criminological inquiry” (p. 307 and 314 respectively; see also: Baer & Chambliss, 1997).

When non-reflexive criminological inquiry relies upon formal definitions of crime—even if circumscribed by “civil and regulatory violations”, as opposed to criminal codes (Kramer, 2020, p. 17)—it comes at the expense of allowing at least one of its variables to be defined by a ragbag of influences: legislators and their ideology, political parties, corporate elites, religious institutions, media portrayals, mythology, and perhaps even the will of constituents (to name but a few). One result of legal formalism is a complex, mutually reinforcing relationship whereby conventional non-reflexive criminological discourse draws its inspiration from a class-biased conceptualization of crime, then in turn legitimizes the very system from which it derives the meaning of that variable (crime); this process occurs to the detriment of cadres of the relatively powerless within society (Lasslett, 2010) and serves to normalize the perpetuation of harm (Falk, 2019). Pemberton (2007) noted that this overly cozy relationship between criminology and the criminal justice system can even epistemologically influence those seemingly most resistant—scholars working from within the critical perspective—in delimiting the nature of crime and therefore the harms that are relevant for consideration.

A means to liberate criminological discourse from the shackles of legal formalism is found in the social harm literature, which analyzes injurious phenomena without unduly (and perhaps unwittingly) acquiescing to the classism embodied in formal legal prohibitions. There are several conceptualizations of social harm prominent in that literature. The Schwendingers (1970) saw an alternative to legal formalism in considerations of morality, which they related to the intrinsic rights due individuals. Rooted in Kant’s second rule of the *Categorical Imperative* (Kant & Paton, 1964), they posited that humans should be viewed as ‘ends in themselves’ that are due equal access to life, health, and freedom from predation and imperialistic elitism (see also: Rawls, 1989). Lasslett (2010) defined social harms as social processes that undermine human existence in delimiting “*moments where the relations, processes and flows of social being disrupt or fail to preserve the structures of organic/inorganic being*” (2010, p. 13; emphasis in original). Johan Galtung (1969) similarly defined harm as the extent to which institutional arrangements impede the ability of individuals to achieve their full human potential.) Many of these scholars suggest that the concept of social harm cannot be divorced from the perceptions of those subject to the harms themselves, in order to prevent legal formalism’s inequitable definition of harm “preordained by the state” (Hillyard & Tombs, 2007, p. 17). They also suggest that a definition of social harm should encompass physical, economic, psychological and cultural harms, addressing broad aspects of social life (e.g., access to education and individual autonomy). Pemberton (2007) suggested that the concept of social harm should be derived from discourse in sociology and social policy, and similar to Lasslett (2010) and Galtung (1969), orient itself towards assessing the degree to which individual and collective needs are addressed.

The social harm scholarship suggests that discourse concerned with harm (as criminology is purported to be) ought to direct attention towards phenomena regardless of its codification by elites. Although such a shift addresses some of the issues precluding analysis of malevolent social movements, it introduces others. Perhaps the most significant concerns its potential to examine phenomena so disparate that it inhibits disciplinary unity, resulting in diminished mutual exclusivity and the malaise of relativism (Hillyard & Tombs, 2007; Lasslett, 2010; Pemberton, 2007) or perhaps engaging in moral crusade (Cohen, 1993, 2001). Pemberton additionally cautions against definitions of social harm that could be co-opted by elites, resulting in “unwarranted interventions” and the phenomenon of ‘net widening’ (2007, p. 37; see also: McMahon, 1990).

## A specification of Edwin Sutherland’s concept of social injury

Discourse structured by legal formalism tends to skew towards analysis of the destructive acts of individuals; however, when one ponders the destruction of millions resulting from malevolent social movements (e.g., the Holocaust), the collective loss that humanity inexorably bears transcends that of individual acts; this occurs by and through structural damage. Modern, twenty-first century Cambodia serves as a potent example of the extent to which genocide can impact structure, and in doing so perpetuate damage exceeding singular acts of violence. More than forty years after Pol Pot and the Khmer Rouge’s murderous campaign of intellectual destruction and forced agricultural collectivization ‘without any of the intervening steps’ (Jackson, 1989), the capital of Phnom Penh still lacks basic services found throughout Southeast Asia: public transportation is limited (although that is changing), healthcare is ranked poorest in the region, electricity is frequently interrupted, and political corruption is normative resulting in severe economic disparity and authoritarian levels of control over dissent (Strangio, 2014). Structurally, educational institutions have struggled- unsurprising given that all Cambodian universities list their date of founding as the early twenty-first century.

Sociological discourse has also generally eschewed analysis of macro-level malevolent social movements (e.g., the Holocaust) and their impact on social institutions, astonishing given that they require mass participation, mobilization of resources, and often coordination of private and public entities. Ontologically, there is little room for debate about the impact that systematic annihilation of at least six million Jewish people, homosexuals, communists, Gypsies, and political dissidents had upon the social milieu of the time. Some scholars have contended that all facets of German society were affected by the silent piecemeal efforts required to systematically annihilate a substantial portion of the population (Aly & Heim, 2002; Rubenstein & Roth, 1987). Historical evidence consistently shows impacts on social organization: from the functioning of families and education to distorting aspects of the economic order. Aly and Heim (2002) found that scholars and advisors responsible for economic planning within Germany were *themselves* highly cognizant that they could not kill all European Jews outright, since they were critical to the functioning of the many institutions throughout Germany and its territories. This makes the subsequent disciplinary myopia in sociology even more astonishing, since an impact on structure was considered even by those who organized and implemented Germany’s brutal enterprise aimed at ‘population redistribution’ (Aly & Heim, 2002).

Given the structural and human harms produced by macro-level malevolent social movements, it is suggested that sociologists and criminologists consider *social injury* in analyzing these largely overlooked phenomena. The concept of social injury is not novel- Edwin Sutherland first invoked it as one of two criteria necessary to define an act as crime (“legal provision of a penalty for the act” being the other; 1945, p. 132). In the article “Reimagining Sutherland”, Simpson (2019) noted that “Sutherland set the standard for empirical research on corporate offenders while providing a critical lens with which to view legal processes and state response to offenders” (p. 190; see also: Sutherland, 1949). However, Sutherland’s formulation reflected his focus on white collar crimes, and he never went on to further develop social injury or consider its utility in understanding phenomena that tends to exist in a jurisprudential hinterland (e.g., genocide). The Schwendingers (1970) critiqued Sutherland’s unsophisticated formulation, lamenting that “he never delineated his criteria of social injury in any organized manner” (p. 126). Despite these shortcomings, there is a precedent of considering phenomena akin to social injury in the discourse on social harm and state-corporate crime that transcends legal formalism and narratives fixated upon individual-level pathologies (Kramer, 2020; Kramer et al., 2002; Michalowski & Kramer, 2006); Michalowski (1985) referenced the concept in describing uncodified yet harmful “analogous forms of social injury” (p. 317). The definition of social injury offered in this paper represents an operational response to the Schwendingers’ critique of Sutherland, in the hopes of reinvigorating a concept that has not yet factored significantly in sociological or criminological discourse.

*Social injury refers to the recursive damage to social structure and human potential through the functional impairment of social institutions*. Phenomena that contribute to social injury are grave such that they cause damage on a macro- and microscopic level, negatively impacting human lives and institutions (e.g., the familial, educational, economic, political, and/or informational dispersing contributors to social life). Thus, social injury concerns the linkage between macroscopic structural damage and the often staggering human costs for those embedded within.

Central to understanding social injury is the concept of social structure, invoked by Marx in 1859 to elucidate connections between the economic modes of production and subjugation of the working classes (Marx, 1901; Porpora, 1989). Durkheim focused on structure in relation to social equilibrium, juxtaposing those relations with explanatory narratives predicated upon individual-level pathologies (i.e., psychological), important in establishing sociology as a science (1933; see also: Pope, 1975). Social injury incorporates some of their foundational ideas in considering the functionality of institutions- that social structure and institutions have an ontological basis, that there exist reciprocal relationships between institutions, and that structure and institutions impact human potential (Durkheim, 1933, 1965; Durkheim & Simpson, 1952; Roche, 2005). Other aspects of this formative scholarship were rejected- the teleological reasoning in Durkheim’s Darwinian rationale for functionalism (Pope, 1975), the notion of the collective conscience (Agassi, 1960; Durkheim, 1965), and “sociological holism” positing social institutions as untamable (Pope, 1975, p. 344; see also: Durkheim, 1933). Furthermore, in contrast with social injury, Pope (1975) noted that Durkheim’s focus on social equilibrium was to the detriment of analysis concerning “institutionally structured disorder, conflict, and coercion” (p. 376).

A ubiquitously accepted definition of social structure has yet to be formulated, the concept having been used disparately to explain regularities in behavior, social facts, human relationships, and the rules governing resource distribution (Porpora, 1989). Blau (1977) defined social structure as “the distributions of a population among different social positions that reflect and affect people's relations with one another. To speak of social structure is to speak of differentiation among people” (p. 28). To Scott (1988), social structure is “produced through the intersection of chains of action and their unintended consequences” which “provided the basic subject matter of sociology” (109–110). And to Galtung (1969) social structure incorporates hierarchically structured “systems of interaction” between actors, ranks and levels which are then studied in relation to equality (pp. 175–176). The concept of social structure employed here parsimoniously incorporates several of these elements- *social structure here refers to the totality of institutions and relations that, when functional, contribute to the sustenance of human potential (the provision of food, shelter, health, education and intellectual development, etc.)*. Institutions are entities that collectively contribute to social structure, defined by Messner and Rosenfeld (1994) as “relatively stable sets of norms and values, statuses and roles, and groups and organizations that regulate human conduct to meet the basic needs of a society” (p. 72). These parsimonious definitions are intended to highlight the linkages between macroscopic aspects of society and human suffering that comprise social injury. This definition of social injury is at odds with the counterintuitive aspects of functionalism, through which Merton (1957) saw crime as functional via its contributions to social cohesion, for example.

Merton (1957) differentiated two functional forms endemic to institutions, defining manifest functions as “those objective consequences for a specified unit (person, subgroup, social or cultural system) which contribute to its adjustment or adaptation and were so intended”, and latent functions as “unintended and unrecognized consequences of the same order”; he further noted that the most “distinctive” discourse concerned counterintuitive, often unintended, observations (i.e., latent functions; p. 69). Notwithstanding Merton’s statement on importance, social injury primarily concerns impairment to manifest function because of discordance between the concept ‘impairment’ and his observation that “through the systematic application of the concept of latent function, therefore, *apparently* irrational behavior may *at times* by found to be positively functional for the group (Merton, 1957, p. 70; emphases in original), particularly troubling considering the human costs associated with social injury. Furthermore, although Merton (1957) thought the consideration of latent functions paradigmatic, they come at the risk of incompatibility with Popper’s (1965) criterion of falsifiability demarcating scientific discourse; where latent functionality precludes the logical possibility of dysfunction, such discourse regresses towards the unfalsifiable (perhaps becoming unscientific). Despite the focus on manifest functionality, social injury still abides by Merton’s general theorem that “*the social functions of an organization help determine the structure (including the recruitment of personnel involved in the structure), just as the structure helps determine the effectiveness with which the functions are fulfilled*” (1957, p. 81; emphasis in original). Additionally, the analysis of latent functions is not wholly precluded, since social injury underscores the extent to which power differentials, organizations, and the structure of the law contribute to disciplinary myopia; thus, there may exist the latent function of social injury’s contribution to incremental epistemic development.

The concept of social injury also draws inspiration from the humanistic aspects of Marxian analyses in considering the structural impact on human potential. Marx saw alienation as detrimental for human intellectual development, which provided “the normative basis for both his critique of capitalism and his conception of communism” (Roche, 2005, p. 336; see also: Marx, 1901; Marx & Engels, 2020; Bendix, 1974). Similarly, social injury often entails dehumanization, which “marks not only those whose humanity has been stolen, but…is a distortion of the vocation of becoming more fully human” (Freire, 2005, p. 44); this recognition of a structural impact on human potential is often lacking in both formal legal definitions of crime and attendant scholarly analyses. Social injury is informed by descriptions of social harm and structural violence by Lasslett (2010) and Galtung (1969), who recognized the importance of structure in curtailing human potential, a situation Freire (2005) referred to as one that “interferes with the individual’s ontological and historical vocation to be more fully human” (p. 55). Social injury thus captures the inseparable connection between social structure and human existence, such that damage to structure incurs costs for humans, and those human costs can recursively transform the very nature of institutions and structure. Freire noted this dialectical interdependence of “world and action” (p. 53) in calling for the liberation of the oppressed:

“Just as objective social reality exists not by chance, but as the product of human action, so it is not transformed by chance. If humankind produce social reality (which in the "inversion of the praxis" turns back upon them and conditions them), then transforming that reality is an historical task, a task for humanity” (2005, p. 51).

Lasslett (2010) recognized these linkages in his consideration of the ontological basis for social harm: “that just as factories, neighbourhoods and cities are reified moments generated by the processes, flows and relations of global capitalism, so too are the millions killed in war, the hundreds of thousands killed and maimed in the workplace, and the fading ozone layer” (p. 11). Social injury similarly concerns “processes, flow and relations” in accounting for the interplay between damage to manifest function and concrete impacts on human life, a dialectic approach that Lasslett called “a robust standard…that limits our analysis to the most serious forms of harmful social practice” (2010, p. 11 and 13 respectively). In this way social injury avoids the ‘fetishized analysis’ of discrete events independent of the ‘processes and flows’ that create them (Lasslett, 2013; Stańczak, 2017). Social injury involves recursive damage between structure and human potential, perpetuating harms that Falk (2019) characterized as “longer term dislocations” (p. 10) that have “caused severe harm in the past, continuing into the present, and threatens to do even greater harm in the future” (p. 6–7).

**Fig. 1:**
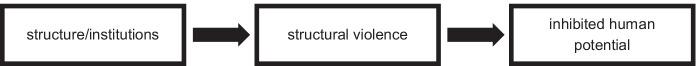
Causal flow of structural violence

Galtung drew attention to these relationships in his article on “structural violence”, which concerned the insidious, persistent, and relatively invisible harms endemic to social structure (e.g., the inequality facilitated and maintained by capitalist neoliberal economic policy; see also: Dilts, 2012). In Galtung’s (1969) formulation, structural violence is a product of the human tendency to create hierarchies, which naturally produces disparities that often go unnoticed- particularly in stable, fixed societies; one outcome is a negative impact on human potential (see Fig. 1). In Winter’s (2012) analysis, Galtung’s structural violence is about the persistence, normalcy (and hence, relative invisibility) of fetters to human potential experienced in everyday life: “poverty, hunger, subordination, and social inclusion”, for example (p. 195); he noted Galtung’s example of “an exploitive caste system or race society” (1969, p. 178). To Vázquez-Arroyo (2012), structural violence refers to persistent and relatively permanent “catastrophes” embedded in the firmament of social structure. Galtung (1969) clarified that “we shall sometimes refer to the condition of structural violence as social injustice” (p. 171), and “the absence of structural violence is what we have referred to as social justice” (p. 183).

**Fig. 2:**
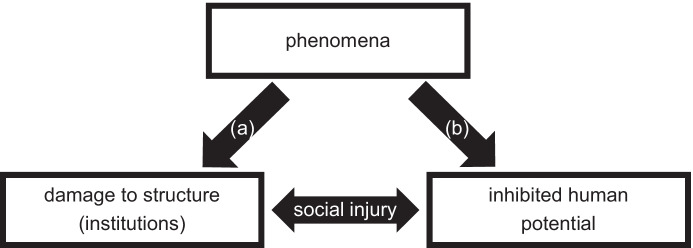
Causal flow of social injury

While there is some congruity between structural violence and social injury, there are several points of divergence. Galtung’s formulation suggests structural violence is “natural”, posing it as tantamount to a foregone conclusion since it is “built into the social structure” (1969; p. 173); in a subsequent revision, he stated that specific preventable events (or sequences of them) can bring about damage to social institutions (Galtung & Höivik, 1971, p. 73). In contrast, social injury is neither natural nor endemic to structure and institutions. It is initiated by some phenomenon (or sequence) and then replicated through the recursive relations between the manifest function of structure and human potential (see Fig. 2).

Another difference between structural violence and social injury is found in Galtung’s differentiation between personal violence “where there is an actor that commits the violence as personal or direct” and structural violence “where there is no such actor” (1969; p. 170). There are indeed actors that can bring about social injury, although their contributions may be diffuse (complicating ascriptions of legal culpability) or normative (challenging sociological ascriptions of deviance); for example, the policymakers attending the *Wannsee Conference* played a critical role in catalyzing the *Final Solution*. Furthermore, Galtung suggests an ontological connection between the two, in that aggregate personal violence can be transformed into structural violence: “when one husband beats his wife there is a clear case of personal violence, but when one million husbands keep one million wives in ignorance there is structural violence” (p. 171). Adding to Galtung, when aggregate personal violence becomes severe such that it recursively impacts structure, it then becomes social injury (since damage to structure will incur future costs to human potential; see: [b] within Fig. 2). However, social injury is not necessarily the product of individual forms of violence in the aggregate, nor is it natural; a singular precipitating phenomenon may be forceful enough to set in motion recursive damage to structure and human potential (e.g., the atomic bombings of Hiroshima and Nagasaki). In other instances, structural violence (in Galtung’s 1971 refinement) can transform into social injury if the relationship between damage to structure and human potential/agency recursively persists (see: [a] within Fig. 2).

The signs that indicate when social injury has occurred were derived from existing scholarly analyses of harmful phenomena. Since social injury concerns the intrinsic connection between human potential and structure, it must consider what Fromm (1964) called “security in the sense that the basic material conditions for a dignified life are not threatened” (p. 32). To Presser (2013), one key to understanding macroscopic harms (e.g., genocide) lie in elucidating atmospheres conducive to violence, and social injury does so in considering damage to manifest function and its impact on human life. Pragmatically, Galtung (1969) proposed a minimalistic quantitative methodology in calculating structural violence, comparing changes to mortality and morbidity rates. Borrowing elements from these scholars, social injury can be assessed using existing metrics on the manifest functions of institutions, comparing them to baseline levels before and after a potential triggering phenomenon, constituting social injury when changes in functionality negatively impact human potential (embodied in, at a minimum, human existence, per Galtung).

Considering who makes this determination is important to prevent repression of the individual through authoritarian conceptualizations of wrongdoing. Hillyard and Tombs (2007) thought the perspective of those subject to the harms no less relevant than that of state agents (who may constitute the perpetrators of harm in certain contexts). Freire (2005) similarly asked “who are better prepared than the oppressed to understand the terrible significance of an oppressive society? Who suffer the effects of oppression more than the oppressed?” (p. 45). However, enabling the harmed to self-recognize social injury presents risk in creating the potential for an unwelcome ‘tyranny of the masses’, debatably as dubious as the ‘tyranny of elites’ intrinsic to legal formalism that has buttressed oppressive policies throughout its existence. Employing the perspectives of the harmed creates an additional conundrum- for how, then, can one differentiate social injury from social progress through revolution (which may cause structural damage and negatively impact human potential). A solution is found in Freire’s (2005) connection between revolutions and oppression; revolutions transform into oppression if they harden into a type of fixed bureaucratic structure, such that “it prevents people from being more fully human” (p. 57). Extrapolating from Freire’s temporal consideration, the revolution that damages structure constitutes social injury when it negatively impacts human potential; revolutions may temporarily appear akin to social injury, but will eventually reveal themselves to be advances in expanding human potential where they are not socially injurious. Where human potential is negatively impacted for lengthy periods of time (i.e., when these aspects of revolution becomes fixed in institutional structures), what was formerly revolution now constitutes social injury.

## A new ‘dark figure’

Criminologists have a history of considering the epistemic implications of their data; Simpson (2019) noted that “one of Sutherland’s most important contributions to the study of corporate crime lies with his critique of official crime data”, in that “the data did not capture crimes by business and, as such, resulted in biased statistics” (p. 190). Official data (e.g., police reports) structured by legal formalism contributes to myopia by only accounting for known codified offenses, remaining ignorant of what is uncodified (i.e., ‘the dark figure of crime’; Coleman & Moynihan, 1996). To Simpson (2019), this “gives scholars an inaccurate and stereotypical image of crime and the typical criminal”, to the detriment of the relatively powerless in society (p. 195). Disciplinary myopia was influenced in some measure by an incongruity between the consideration of disparate harms and the subjective, political, economic, ideological, and hegemonic influences on legal formalism; the Holocaust was not a crime to the *National Socialists* because they did not codify it as such, in much the same way that the American genocide against native peoples was legally eschewed in its time.

The social harm literature and social injury point towards a new, reconfigured and expanded ‘dark figure of harm’, because these phenomena are obscured by: a) typically residing external to formal legal definitions of crime; b) operating within the context of legal systems that are incapable of mounting adequate responses, given the breadth of complicity and structural contributors to harm; c) often involving otherwise normal individuals and conforming behavior that defies existing theoretical explanations, particularly when shaped by legal formalism.

## Discussion

It is an absurdity that criminological and sociological discourse recognizes the connections between structure and crime but generally eschews consideration of the structural damage and human costs emanating from malevolent social movements. This is lamentable considering the scale of atrocities like the Holocaust, even more so considering the estimated 100,000,000 deaths caused by a panoply of wars and genocides throughout the twentieth century (Adams & Balfour, 1998; Bauman, 1988; Brannigan & Hardwick, 2003; Eliot, 1972). A systematic examination of socially injurious phenomena holds the potential of contributing much to sociological and criminological discourse- assessing the limitations of foundational constructs like *mala in se, actus rea*, *mens rea*, and deviance; probing the moral boundaries of latent functional analysis; and exploring the incompatibility of legal formalism and scientific discourse, to name but a few. The adoption of standards like ‘social harm’ and ‘social injury’ overcome some of the impediments created by legal formalism, but not without some of the shortcomings previously considered. The specification of Sutherland’s concept of social injury suggested here is intended to highlight infrequently considered harmful phenomena, and in doing so liberate criminological and sociological discourse from the constraints of legal formalism.

This phenomenon of social injury suggests several directions for additional research. On a pragmatic level, the focus on harms emanating from recursive linkages between structure and individuals suggests inquiry into the conditions whereby those connections can be broken (or forestalled); an examination of prior socially injurious phenomena may elucidate the conditions under which social injury previously diminished or ceased. Social injury also calls for inquiry into the most appropriate means of reconstructing institutions in order to promote the expansion of human potential; similarly, scholarly attention may turn towards assessing ways to mitigate the negative impact of social injury on human lives. Social injury also raises a number of philosophical and theoretical questions. Merton (1957) suggested a moral boundary to latent function analysis, because “moral evaluations in a society tend to be largely in terms of the manifest consequences of a practice or code, we should be prepared to find that analysis in terms of latent functions at times runs counter to prevailing moral evaluations” (p. 74). This is particularly problematic regarding analysis of the Holocaust—which caused deeply significant harm to the Jewish people—necessitating ethical considerations related to the appropriateness of discourse (Bernard-Donals & Glejzer, 2001). Social injury also calls for renewed consideration of existing theories and concepts to better understand connections between social change and harm (e.g., economic change, political revolution, etc.), which calls for incorporating the temporal dimension within “historically grounded theories of long-term social processes” (Aminzade, 1992, p. 475).

Social injury also establishes a framework for the consideration of harmful phenomena other than genocide. Commencing after the 1912 *Hague International Opium Convention* and accelerating thereafter, the global ‘war on drugs’ has cost millions of lives, consumed vast resources from almost all nations of the world, and served as a distraction from more serious social harms (Baum, 1996). In the Western world—where it was rationalized via ideologically driven statements of dubious intellectual merit (e.g., moral panics on ‘reefer madness’ [Zimmer & Morgan, 1997] and ‘crack babies’ [Coles, 1993])—whole areas of social life have been impacted- from systematic disinvestment in communities of color to the legitimization of militaristic police forces to the social, economic, intellectual and political disenfranchisement of those subject to drug law persecution (Reiman & Leighton, 2017); the effects of these campaigns in the Southern hemisphere have been no less catastrophic (Carpenter, 2003). In America, where drug convictions can preclude one’s access to student financial assistance, swaths of the population have been disqualified from higher education and the meaningful contributions to society for which it is a prerequisite (Lovenheim & Owens, 2014). Despite recent movements to curtail some policies, many nations have unwittingly replicated these dynamics (imposing drug laws that impact the economy, polity and education structures). An example is found in modern Japan and its draconian *Cannabis Control Act*, a policy not derived from Japanese custom, but imposed at the close of WWII by occupying American forces who based it on flawed early- to mid-twentieth-century rationalizations against the use of psychoactive substances (Mitchell, 2014).

The construct of social injury is not constrained by legal formalism in acknowledging the harmful actions of governments. As the Covid-19 pandemic swept the globe in 2020, political elites in the US spoke and acted in defiance of scholarship on the spread of infectious diseases, influencing segments of the population to question the validity of scientific research. Despite lethargic steps to quell the spread of disease, within three months legislation was proposed indemnifying private business from pandemic related claims (Tankersley & Savage, 2020). Within a year, more than half a million people would succumb in the US alone, causing transformations to the economy and education system, in addition to human costs borne by families whose lives were upended by the death of a loved one. This was neither inevitable nor the case in other countries; in New Zealand, public officials acted swiftly to protect public health by taking action to quell the spread of disease (Cave & Solomon, 2020). Another illustration lie in the American war on terror as reified in Iraq and Afghanistan, which represented no crime to the American government but severely impacted the structure of society in those countries, resulting in widespread poverty and dislocation. In the case of Iraq, the ouster of Saddam Hussein caused social injury through recursive damage to political structure and human potential, exacerbated through the creation of a power vacuum filled by ISIS.

Where does social injury fits within current disciplinary boundaries? The connections between legal formalism and criminology suggest it is perhaps an ill-suited venue; furthermore, Lasslett (2010) noted that mere invocation of the concept ‘crime’ perpetuates the myth that it intrinsically concerns harm. Temporal considerations perhaps situate social injury within ‘historical sociology’, which concerns “sequences of events that have unfolded in similar but not identical fashion in a variety of different historical contexts” (Aminzade, 1992, p. 458). Additionally, analysis of socially injurious phenomena may already occur in other disciplines, for example political science (although its analysis of the Holocaust is lacking; see: King, 2012). Other scholars have suggested the creation of new disciplines and attendant publications specifically attuned to examining harm without acquiescing to the epistemic restrictions of legal formalism (e.g., social harm studies and *State Crime Journal*). Harm on the scale of nation states challenges existing legal codes and scholarly analyses shaped by them. Social injury transcends these challenges through examination of recursive harm to structure and human potential, in the hope of developing a more nuanced understanding of these frequently overlooked phenomena.

## Data Availability

not applicable
